# Violent actions against children

**DOI:** 10.1016/j.dib.2017.04.026

**Published:** 2017-05-02

**Authors:** Muhammad Alhammami, Chee-Pun Ooi, Wooi-Haw Tan

**Affiliations:** Multimedia University, Malaysia

## Abstract

We present in this paper a novel dataset (MMU VAAC) for violent actions against children recognition. This original dataset has been recorded using Microsoft Kinect with the usage of a child mannequin. MMU VAAC dataset contains skeleton joints, depth, and RGB modalities.

## **Specification Table**

**Table:** 

Subject area	*Computer vision*
More specific subject area	*Human action recognition*
Type of data	*Table of skeleton joints.csv file, depth images, RGB images*
How data was acquired	*Controlled recording*
Data format	*Raw and Processed*
Experimental factors	*Indoors, using Microsoft Kinect sensor and child mannequin*
Experimental features	*Data mining was performed after dataset capturing.*
Data source location	*Digital Home Lab, Faculty of Engineering, Multimedia University, Cyberjaya, Malaysia*
Data accessibility	*Raw skeleton joints modality is attached to this paper.*

## **Value of the data**

• The recognition of violence against children is a critical application in the field of human action recognition which still has not gain any interest [1].
• There are no previous efforts to recognize physical abuses against children.
• The main difficulty in capturing a dataset of child abuses is that we cannot let any child face any abuse. We cannot also install cameras in schools and houses without permission of authorities. There are also not enough recorded cases of child abuses on YouTube.
• All these aspects motivated us to record a novel dataset of physical abuses against children but using a child mannequin.
• Now, using this dataset, researchers and developers can instantly analyze the composition of abuse activities [2] to immediately start developing prototypes for systems which recognize violence against children using computer vision techniques [3].

## 1 Data

**Fig. 1:**
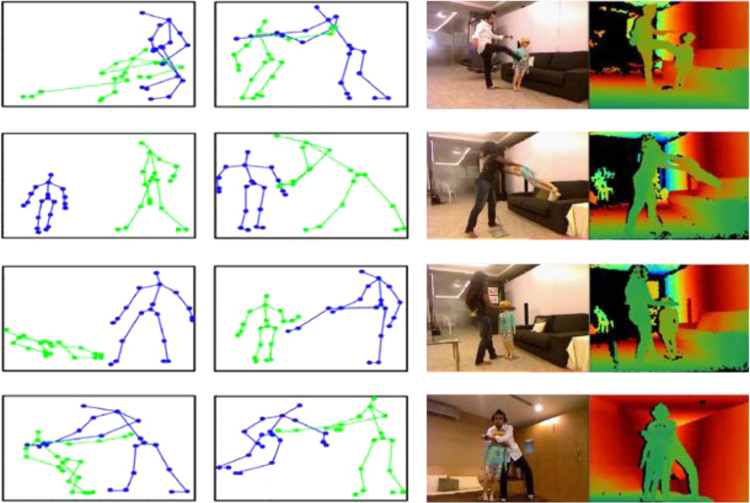
Shots of RGB-D modalities and representation of skeleton joints of MMU VAAC dataset.

The VAAC dataset contains RGB, depth and skeleton data of all recorded scenes. RGB-D images carry files׳ names correspondence to the frame stamp from the device. The skeleton joints file contains the following attributes: joints coordinates in 3D space (*x*,*y*,*z*) for both the adult actor and the child and their IDs besides the frame time stamp and the mannequin (Fig. 1).

The recorded actions contain:

• Violent actions: kicking, punching, throwing, shoving, strangling, and slapping.
• Nonviolent actions: touching, hugging, lifting, laying down, etc.

## 2 Experimental design, materials, and methods

The MMU VAAC database was recorded with the utmost care and attendance by the author. For avoiding effects of any errors and omissions occurred, all recorded scenes were subject to later inspection while doing the preprocessing phase. All actions were acted and recorded in an indoor environment the living room and bedroom of Digital Home Lab at the faculty of engineering in Multimedia University, Malaysia. The sensor used is Microsoft Kinect placed frontal-parallel to the subjects and the child mannequin. It was mounted at the height of 90 cm while the distance from the sensor to the subject was 190 cm. The effective range of the Kinect sensor is 1.2–3.5 m. The sensor motorized pivot can tilt the sensor up to 27° either up or down while has an angular field of view of 57° horizontally and 43° vertically. The horizontal field of the Kinect sensor at the minimum viewing distance of ~0.8 m (2.6 ft.) is, therefore, ~87 cm (34 in.), and the vertical field is ~63 cm (25 in.). Hence, the resulted resolution is over 1.3 mm (0.051 in.) per pixel. During the acquisition period, a decision was made to acquire data at the highest resolution and frame rate possible to minimize the effect of noise from the environment or the sensor, so it recorded depth and color frames with 640×480 resolution and speed of 30 frames per second. The child mannequin size is 65 cm, and actors׳ size has the range of 150–170 cm. The players took the freedom to stand and perform the actions spontaneously. However, they were asked to use both hands and legs. The subjects have been invited to help calibrate the Kinect camera before the start of the video recording. This was done by standing in front of the Kinect camera with arms spread out wide for the joints of the subjects to be detected.

The acquisition was done over a period of five days and involved 4 participants. The subjects were aged between 20 and 25 years. Lighting conditions were uncontrolled to achieve a more realistic set of data. There was one station only which contains the Kinect sensor. The station installed once in the living room and once in the bedroom. All dataset was recorded and stored on a hard disc.

**Fig. 2:**
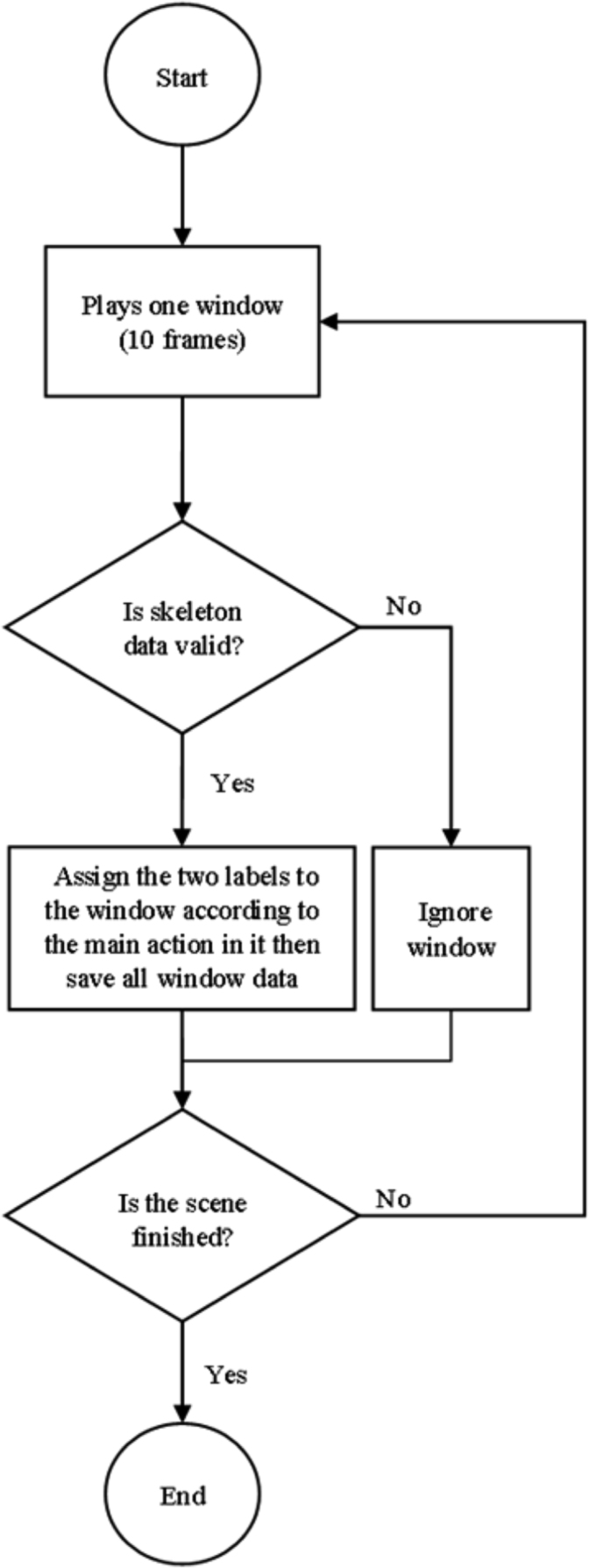
Segmentation process of the recorded scenes.

For skeleton joints modality, the following routine was used for creating the data set Fig. 2. We manually segmented all the scenes we have about the eight actions. Moreover, we assigned each sliding window to one action depending on the main activity in each sliding window. Here, we defined and included two more actions (approaching and departing) to the original actions for better and more accurate assigning each window to one main action.
