# Supplementary material for: Violent actions against children
Source: Data Brief. 2017 May 2;12:480–4. doi: 10.1016/j.dib.2017.04.026 (PMC5423304; doi:10.1016/j.dib.2017.04.026)
Supplement: Supplementary file 1 — Supplementary material [file mmc1.rtf]

AUTHOR DECLARATION
We wish to draw the attention of the Editor to the following facts which may be considered as
potential conflicts of interest and to significant financial contributions to this work. [OR]
We wish to confirm that there are no known conflicts of interest associated with this
publication and there has been no significant financial support for this work that could have
influenced its outcome.
We confirm that the manuscript has been read and approved by all named authors and that
there are no other persons who satisfied the criteria for authorship but are not listed. We
further confirm that the order of authors listed in the manuscript has been approved by all of
us.
We confirm that we have given due consideration to the protection of intellectual property
associated with this work and that there are no impediments to publication, including the
timing of publication, with respect to intellectual property. In so doing we confirm that we
have followed the regulations of our institutions concerning intellectual property.
We understand that the Corresponding Author is the sole contact for the Editorial process
(including Editorial Manager and direct communications with the office). He/she is
responsible for communicating with the other authors about progress, submissions of
revisions and final approval of proofs. We confirm that we have provided a current, correct
email address which is accessible by the Corresponding Author and which has been
configured to accept email from (dr.mhammami@outlook.com). 

Corresponding Author
Muhammad Alhammami
